# The Role of Trabectedin in Soft Tissue Sarcoma

**DOI:** 10.3389/fphar.2022.777872

**Published:** 2022-02-23

**Authors:** Tomoki Nakamura, Akihiro Sudo

**Affiliations:** Departmemt of Orthopaedic Surgery, Mie University Graduate School of Medicine, Tsu, Japan

**Keywords:** trabectedin, soft tissue sarcoma, clinical trials, progression-free survival, advanced soft tissue sarcoma

## Abstract

**Background:** Systemic chemotherapy for advanced disease is another therapeutic option in the management of metastases in soft tissue sarcoma (STS). Doxorubicin either alone or in combination with ifosfamide has been used as first-line chemotherapy. Furthermore, in the past decade, new drugs have been shown to be effective in the treatment of advanced STS after the failure of first-line anthracycline-based chemotherapy: trabectedin, pazopanib and eribulin. However, the appropriate usage of these agents has not been established.

**Methods:** We summarized clinical trials of trabectedin focusing on the efficacy and toxicity of trabectedin in the treatment of STS.

**Results:** Trabectedin can be administered safely and effectively to the patients with advanced STS at second line setting or later. Although trabectedin may be effective as first-line treatment in selected patients, anthracycline-based chemotherapy should be recommended because no regimen in addition to trabectedin has proved to be unequivocally superior to doxorubicin as the first-line treatment for locally advanced or metastatic STS. Nucleotide excision repair (NER) and homologous recombination (HRe) repair may be of particular importance as efficacy of trabectedin.

**Conclusion:** Trabectedin has shown a favorable toxicity profile and is an alternative therapeutic option in patients with advanced STS.

## Introduction

Soft tissue sarcoma (STS) is a rare, heterogeneous group of tumors (Clark et al., 2005; Bourcier et al., 2019). The incidence of STS is fewer than six per 100,000 cancer cases, which represents 1*–*2% cases of all cancer in adults (Clark et al., 2005). Lung metastasis from STS occur in 20–50% of these patients (Nevala et al., 2019; Nakamura et al., 2021). Metastasectomy is the standard treatment for improving survival in patients with lung metastasis from STS (Marulli et al., 2017; Nakamura et al., 2017; Stamenovic et al., 2021). Recently, radiofrequency ablation (RFA) of the lung has also proved to be a useful option which promise a similar outcome to metastasectomy (Nakamura et al., 2009; Nakamura et al., 2017; Tetta et al., 2021). However, even after a seemingly complete resection of metastatic tumors, metastasis will recur in 40–80% of the patients (Weiser et al., 2000). Systemic chemotherapy for advanced disease is another therapeutic option in the management of metastases (Bramwell et al., 2003; Judson et al., 2014; Ratan and Patel, 2016; Smrke et al., 2020). Doxorubicin either alone or in combination with ifosfamide has been used as first-line chemotherapy (Judson et al., 2014; Smrke et al., 2020). Furthermore, in the past decade, new drugs have been shown to be effective in the treatment of advanced STS after the failure of first-line anthracycline-based chemotherapy: trabectedin, pazopanib and eribulin. However, the appropriate usage of these agents has not been established because of the rarity of STS and difficulty of large study.

Trabectedin is a synthetic, marine-derived anticancer alkaloids derived from the Caribbean tunicate, Ecteinascidia turbinate (Carter and Keam, 2007; Cuaves and Francesch, 2009). The success of trabectedin in preliminary clinical trials for STSs has led to the approval of the drug in European countries in 2007 for the treatment of patients with advanced STS after the failure of therapy with doxorubicin either alone or in combination with ifosfamide (European Medical Agency). In 2015, Food and Drug Administration (FDA) approved trabectedin for the treatment of patients with unresectable or metastatic liposarcoma or leiomyosarcoma who received a prior anthracycline-containing regimen (Barone et al., 2017). Approval was based on the results of a randomized phase III study (ET743-SAR-3007, ClinicalTrials.gov Identifier; NCT01343277) comparing the safety and efficacy of trabectedin 1.5 mg/m2 as a 24-h continuous intravenous (IV) infusion once every 3 weeks with dacarbazine 1,000 mg/m2 IV once every 3 weeks (Demetri et al., 2016). Furthermore, in 2015, trabectedin was approved in Japan for the treatment of patients with STS after a clinical trial targeting translocation-related sarcoma (TRS) (Kawai et al., 2015).

Although the detailed indication of trabectedin is different in the world, several studies were conducted for finding the characteristics of trabectedin in the field of STS. The purpose of this review is to summarize the efficacy and toxicity of trabectedin in the treatment of STS.

### How Does Trabectedin Worked (Figure.1)?

Trabectedin is a tetrahydroisoquinoline alkaloid derived from the Caribbean marine tunicate, Ecteinascidia turbinata, and is currently produced synthetically (Carter and Keam, 2007; Cuaves and Francesch, 2009). Trabectedin interacts with the minor groove of DNA double helix and alkylates guanine at the N2 position, which bends toward the major groove (D’Incalci and Galmarini, 2010; D’Incalci et al., 2014; Larsen et al., 2016), triggering a cascade of events that interferes with several transcription factors, DNA binding proteins, and DNA repair pathways, resulting in a delayed S phase progression and accumulation of cells in G2 phase and ultimately apoptosis (D’Incalci and Galmarini, 2010). Furthermore, the pattern of sensitivity observed in cells deficient in DNA damage repair (DDR) mechanisms is different. In the case of trabectedin, nucleotide excision repair (NER) and homologous recombination (HRe) repair are of particular importance (Soares et al., 2007; Italiano et al., 2011; Schoffski et al., 2011; Laroche-Clay et al., 2015). In contrast to other DNA-damaging agents such as cisplatin, NER-deficient cells are two to ten times less sensitive to trabectedin (Damia et al., 2001; Brodowicz 2014). On the other hand, cells deficient in HRe repair are sensitive to trabectedin (Avila-Arroyo et al., 2015). Therefore, DDR-related genes might be potential predictive biomarkers for this drug. Trabectedin seems to be more active in the context of high levels of expression of NER gene (ERCC1 and ERCC5) and low expression levels of HRe genes (BRCA1). Trabectedin selectively targets monocytes and tumor associated macrophages and downregulates the production of inflammatory mediators such as IL-6 and CCL2, which may underlie the strong association between chronic inflammation and cancer progression (D’Incalci et al., 2014; Germano et al., 2013).

**FIGURE 1:**
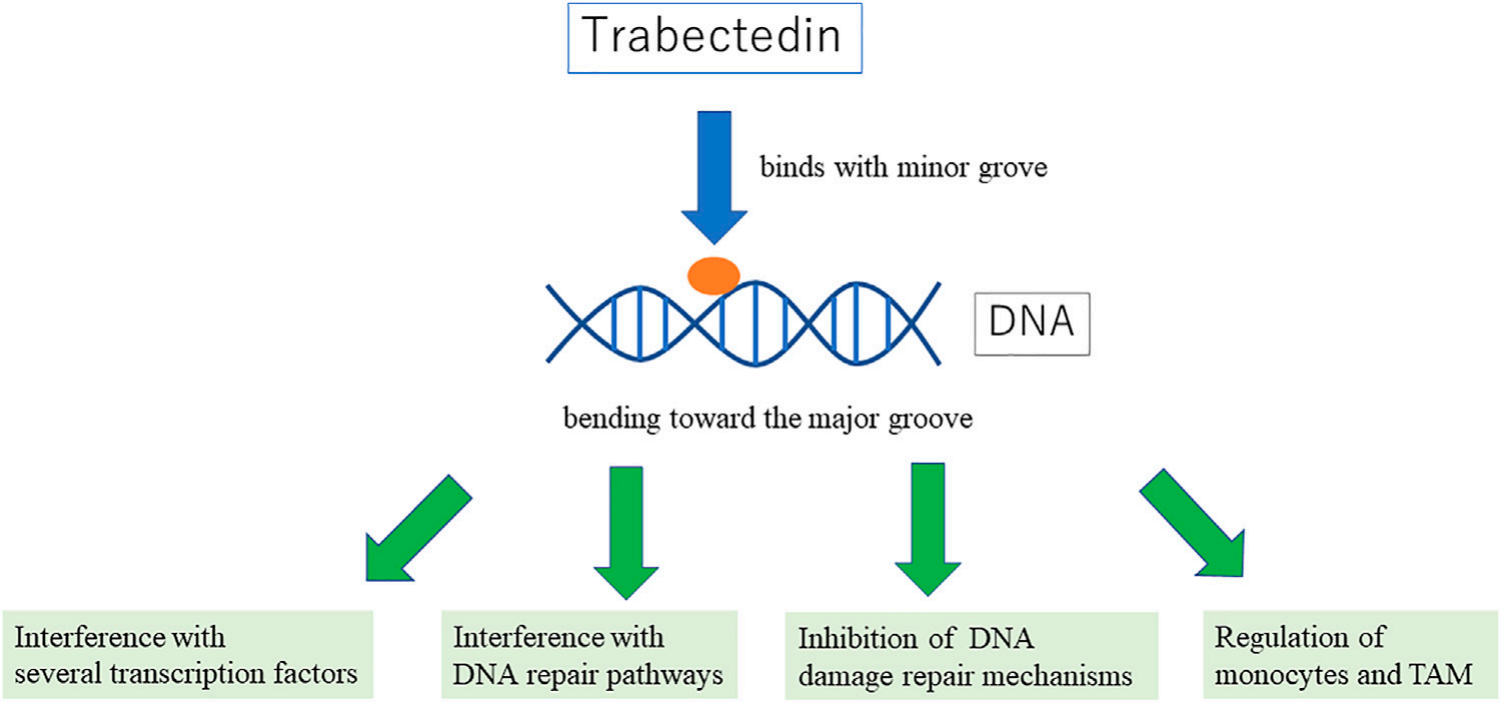
showing the mechanism of trabectedin.

Trabectedin also has a specific mechanism against some translocation-related sarcomas. Trabectedin blocks the *trans*-activating ability of chimaeras by displacing the oncogenic fusion protein FUS-CHOP from its target promoters in myxoid liposarcoma (Di Giandomenico et al., 2014; Forni et al., 2009). Recently, Genomic analysis in murine models of human myxoid liposarcoma showed that prolonged treatment causes losses in 4p15.2, 4p16.3 and 17q21.3 cytobands leading to acquired-resistance against trabectedin (Mannarino et al., 2021).

### Trabectedin Treatment After the Failure of Prior Chemotherapy in Advanced STS (Table 1)

Two phase II trials in 2004 provided the initial analysis of trabectedin in STSs (Garcia-Carbonero et al., 2004; Yovine et al., 2004). Trabectedin was administered at a dose of 1.5 mg/m^2^, 24-h IV infusion every 3 weeks. The first of these studies was conducted in 54 advanced or metastatic STS patients with failure of prior chemotherapy (Yovine et al., 2004). The objective response rate was 4%, although the disease control rate at 6 months was 24%. The median progression-free survival (PFS) and overall survival (OS) were 1.9 and 12.8 months, respectively. The second phase II trials reported a response rate of 8% in 36 recurrent or metastatic STS patients with disease progression despite prior chemotherapy (Garcia-Carbonero et al., 2004). The median PFS and OS were 1.7 and 12.1 months, respectively.

**TABLE 1:** clinical trials for advanced STS.

Study design/Trial registration	Setting	Patients	Regimen	ORR (%)	PFS (mos)	OS
Phase 2 (Yovine et al., 2004)	the second-line setting or later	STS (n = 54)	A. T 1.5 mg/m2 24-h IV q3ws	4.0	1.9	12.8 months
Phase 2 (Garcia-Carbonero et al., 2004)	the second-line setting or later	STS (n = 36)	A. T 1.5 mg/m2 24-h IV q3ws	8.0	1.7	12.1 mos
Phase 2 (Demetri et al., 2009)	the second-line setting or later	LPS or LMS (n = 270)	A. T 1.5 mg/m2 24-h IV q3ws	5.6	3.7	13.9 months
R (1:1)			B. T 0.58 mg/m2 3-h IV weekly	1.6	2.3	10.8 months
Phase 2 (Kawai et al., 2015)	the second-line setting or later	TRS (n = 76)	A. T 1.2 mg/m2 24-h IV q3ws	11.0	5.6	Not reached
R (1:1)			B. Best supportive care	0.0	0.9	8 months
JapicCTI-121850						
phase 3 (Demetri et al., 2016; Patel et al., 2019)	the second-line setting or later	LPS or LMS (n = 518)	A. T 1.5 mg/m2 24-h IV q3ws	9.9	4.2	13.7 months
R (2:1)			B. Dac 1 g/m2 20- to 120-min q3ws	6.9	1.5	13.1 mos
NCT01343277						
phase 3 (Le Cesne et al., 2021)	the second-line setting or later	STS (n = 103)	A. T 1.5 mg/m2 24-h IV q3ws	13.7	3.1	13.6 months
R (1:1)			B. Best supportive care	0.0	1.5	10.8 months
NCT02672527						
phase 3 (Blay et al., 2014)	the first-line setting	TRS (n = 121)	A. T 1.5 mg/m2 24-h IV q3ws	5.9	16.1	
R (1:1)			B. D 75 mg/m2 q3ws or D 60 mg/m2 + I 6–9 g/m2 q3ws	27.0	8.8	
NCT00796120						
phase 2 (Bui-Nguyen et al., 2015)	the first-line setting	STS (n = 133)	A. T 1.5 mg/m2 24-h IV q3ws	4.7	3.1	
R (1:1:1)			B. T 1.3 mg/m2 3-h q3ws	14.8	2.8	
NCT01189253			C. D 75 mg/m2 q3ws	25.6	5.5	
phase 2 (Martin-Broto et al., 2016)	the first-line setting	STS (n = 115)	A. T 1.1 mg/m2 3-h plus D 60 mg/m2 q3ws		5.5	13.3 months
R (1:1)			B. D 75 mg/m2 q3ws		5.7	13.7 months
NCT01104298						
phase 2 (Grosso et al., 2020)	the first-line setting	STS (n = 24)	A. T 1.3–1.5 mg/m2 24-h IV q3ws		4	12 months
NCT02066675						
phase 2 (Pautier et al., 2015; Pautier et al., 2021)	the first-line setting	STS (n = 62)	T 1.1 mg/m2 3-h plus D 60 mg/m2 q3ws		12.9	38.7 months
NCT02131480						

Abbreviations: R, randomized study; STS, soft tissue sarcoma; A, group A; B, group B; C, group C; T, trabectedin; D, doxorubicin; I, ifosfamide; Dac, dacarbazine; q3ws, every 3 weeks; h, hour; ORR, objective response rate according to Response Evaluation Criteria In Solid Tumors criteria; PFS, progression-free survival; OS, overall survival; mos, months; LPS, liposarcoma; LMS, leiomyosarcoma; TRS, translocation-related soft tissue sarcoma.

In addition to the efficacy of trabectedin 1.5 mg/m^2^ 24-h IV infusion every 3 weeks, a weekly trabectedin schedule (0.58 mg/m^2^ 3-h IV infusion for 3 consecutive weeks in a 4-weeks cycle) was demonstrated to have substantial anticancer activity in pretreated ovarian cancer (Krasner et al., 2007). To assess the efficacy and safety of these two schedules in STS, a randomized, open-label, phase II trial was conducted in patients with advanced and/or metastatic liposarcomas or leiomyosarcomas after the failure of standard therapies (Demetri et al., 2009). The time to progression was the primary endpoint. The 24-h IV q3ws demonstrated a superior time to progression of 3.7 vs 2.3 months (hazard ratio (HR), 0.734; 95% confidential interval (CI), 0.554–0.974; *p* = 0.0302). The median PFS was 3.3 vs 2.3 months (HR, 0.755; 95% CI, 0.574–0.992; *p* = 0.0418). The median OS was 13.9 vs 11.8 months (HR, 0.843; 95% CI, 0.653–1.090; *p* = 0.1920). After these results, trabectedin 1.5 mg/m2 24-h IV infusion every 3 weeks is common schedule of trabectedin treatment.

A recent phase II study in the second-line setting or later has been reported (Kawai et al., 2015). This study was a randomized phase II study of trabectedin monotherapy vs best supportive care (BSC) in patients with translocation-related sarcoma subtypes. The patients were randomized (1:1) to receive trabectedin (1.2 mg/m2 24-h IV infusion every 3 weeks) or best supportive care. The trabectedin dose of this trial was 1.2 mg/m^2^ according to the results of a phase I study in Japanese patients with STSs, in which two of three patients had dose-limiting toxicity at 1.5 mg/m^2^ (Ueda et al., 2014). The primary endpoint of this trial was the PFS. The median PFS of the trabectedin group was 5.6 months and that of the BSC group was 0.9 months (HR, 0.07; 95% CI, 0.03–0.16; *p* < 0.0001). The success of trabectedin in this clinical trial for STSs has led to the approval of the drug in Japan.

In 2015, trabectedin has been approved by the FDA based on the result of an open-label, randomized (2:1) phase III trial of trabectedin (n = 345) vs dacarbazine (n = 173) in patients with metastatic liposarcoma or leiomyosarcoma (ET743-SAR-3007, ClinicalTrials.gov Identifier; NCT01343277) (Demetri et al., 2016). In the final analysis of PFS, trabectedin administration resulted in a 45% reduction in the risk of disease progression or death compared with dacarbazine. The median PFS was 4.2 vs 1.5 months (HR, 0.55; 95% CI, 0.44–0.70; *p* < 0.001).

After the analysis of PFS in 2016, the final overall survival (OS) results in an open-label, randomized (2:1) phase III trial of trabectedin (n = 384) vs dacarbazine (n = 193) in 577 patients with metastatic liposarcoma or leiomyosarcoma (ET743-SAR-3007, ClinicalTrials.gov Identifier; NCT01343277) was published in 2019 (Patel et al., 2019). Despite improved disease control by trabectedin, no improvement in OS was observed. The median OS for trabectedin and dacarbazine was 13.7 and 13.1 months, respectively (*p* = 0.49). Trabectedin prolonged time to starting any post-study anticancer therapy in the trabectedin arm (median 6.8 months) compared with the dacarbazine arm (3.5 months).

As a subgroup analysis of phase III study (ET743-SAR-3007, ClinicalTrials.gov Identifier; NCT01343277), 131 elderly patients were collected for evaluating the safety and efficacy in elderly patients with metastatic liposarcoma or leiomyosarcoma (Jones et al., 2018). Among 131 patients (trabectedin = 94; dacarbazine = 37), elderly patients treated with trabectedin (median age = 69 years) showed significantly improved PFS (4.9 versus 1.5 months, respectively; HR 0.40; *p* = 0.0002) but no significant improvement in OS (15.1 vs 8.0 months, respectively; HR = 0.72, *p* = 0.18). The safety profile for elderly trabectedin-treated patients was comparable to that of the overall trabectedin-treated study.

The French Sarcoma Group assessed the efficacy, safety, and quality of life of trabectedin versus BSC in patients with advanced STS (ClinicalTrials.gov Identifier; NCT02672527) (Le Cesne et al., 2021). This study was a randomized phase III study. The patients were randomized (1:1) to receive trabectedin (1.5 mg/m^2^ 24-h IV infusion every 3 weeks) or BSC. The primary endpoint of this trial was the PFS. The median PFS of the trabectedin group (n = 52) was 3.1 months and that of the BSC group (n = 51) was 1.5 months (HR, 0.39; 95% CI, 0.24–0.64; *p* < 0.0001). Trabectedin demonstrates superior disease control to BSC. In this study, the health-related quality of life (QOL) was assessed using the 30-item core European Organization for the Research and Treatment of Cancer (EORTC) Quality-of-Life Questionnaire (EORTC QLQ-C30). Compliance to EORTC QLQ-30 was good in both arm at baseline and after 8 months decreased to 59% in the trabectedin arm and 63% in the BSC arm. Therefore, trabectedin demonstrated superior disease control to BSC without impairing QOL.

### Clinical Trial as First-Line Chemotherapy in Advanced STS

Generally, trabectedin is considered to be administered for the patients with advanced STS after the failure of first-line chemotherapy. Some clinical trials aimed to develop the trabectedin treatment as first-line chemotherapy. One phase III study in the first-line setting has been reported (ClinicalTrials.gov Identifier; NCT00796120). (Blay et al., 2014). This study was a randomized, phase III study of first-line trabectedin vs doxorubicin-based chemotherapy in patients with TRS subtypes. The primary endpoint was PFS. Patients were randomized (1:1) to receive trabectedin (1.5 mg/m^2^ 24-h IV infusion every 3 weeks), doxorubicin (75 mg/m^2^ IV every 3 weeks), or doxorubicin (60 mg/m^2^ IV) plus ifosfamide (range, 6–9 g/m^2^ IV) every 3 weeks. There was no difference in the median PFS or OS between the groups (*p* = 0.9573 and *p* = 0.3659, respectively). The response rate according to the RECIST (Response Evaluation Criteria In Solid Tumors) criteria was significantly higher in the chemotherapy arm (27%) compared to the trabectedin arm (5.9%). In contrast, the response rate according to the Choi criteria showed fewer differences between the chemotherapy arm (45.9%) and trabectedin arm (37.3%).

Recently, results from randomized, multicenter, prospective dose-selection phase IIb trials to evaluate whether trabectedin as first-line chemotherapy for advanced/metastatic STS prolongs the PFS, compared to doxorubicin, were published (ClinicalTrials.gov Identifier; NCT01189253) (Bui-Nguyen et al., 2015). One hundred and thirty-three patients were randomized (1:1:1) to doxorubicin, trabectedin (3-h [T3h arm] infusion every 3 weeks), or trabectedin (24-h [T24h arm] infusion every 3 weeks). The median PFS was 2.8 months in the T3h arm, 3.1 months in the T24h arm, and 5.5 months in the doxorubicin arm. No significant improvement in the PFS was observed in the trabectedin arms as compared to the doxorubicin arm (T24h vs doxorubicin: HR 1.13; 95% CI 0.67–1.90, *p* = 0.675; T3h vs doxorubicin: HR 1.50, 95% CI 0.91–2.48, *p* = 0.944).

Spanish group conducted randomized, phase II clinical trial for comparing the clinical outcome of trabectedin plus doxorubicin with doxorubicin as first line treatment of advanced STS (ClinicalTrials.gov Identifier; NCT01104298) (Martin-Broto et al., 2016). The primary endpoint was PFS. One hundred and fifteen patients were randomized (1:1) to trabectedin (1.1 mg/m2 in a 3-h infusion) plus doxorubicin (60 mg/m2) as the experimental arm or doxorubicin (75 mg/m2) as control arm. PFS was 5.5 months in the control arm and 5.7 months in the experimental arm (HR, 1.16; 95% CI, 0.79–1.71, *p* = 0.45). The proportion of patients with grade 3 or 4 thrombocytopenia, asthenia, and liver toxicity was significantly higher in the experimental arm. Trabectedin plus doxorubicin did not show superiority over doxorubicin alone as first-line treatment of advanced STS.

Italian Sarcoma Group reported a phase II single-arm study for investigating trabectedin as a first-line treatment in elderly patients with advanced STS who were inoperable and were unfit to receive standard anthracycline-based chemotherapy (TR1US study, ClinicalTrials.gov Identifier; NCT02066675) (Grosso et al., 2020). The primary endpoint was PFS at 3 months and the rate of clinically limiting toxicities (CLTs). With a median age of 79 years, 24 patients were enrolled. progression-free survival at 3 months was 71%. Median PFS and OS were 4 and 12 months, respectively. There were no significant differences in trabectedin pharmacokinetics compared with younger populations.

Although trabectedin may be effective as first-line treatment in selected patients, anthracycline-based chemotherapy should be recommended because no regimen in addition to trabectedin has proved to be unequivocally superior to doxorubicin as the first-line treatment for locally advanced or metastatic STS (Seddon et al., 2017).

Interestingly, French Sarcoma Group performed a single-arm, multicentre, phase II study (LMS-02, ClinicalTrials.gov Identifier; NCT 02131480) of doxorubicin combined with trabectedin as first-line treatment in patients with uterine leiomyosarcoma and STS (Pautier et al., 2015; Pautier et al., 2021). Patients received 60 mg/m2 IV doxorubicin followed by trabectedin 1.1 mg/m2 as a 3 h infusion on day 1 and pegfilgrastim on day 2, every 3 weeks, up to six cycles. Median PFS in 62 patients with STS was 12.9 months (95%CI 9.2–14.1 months). The median OS was 38.7 months (95%CI 31–52.9 months). Now, LMS04 trial (ClinicalTrials.gov Identifier; NCT02997358), a randomized phase III study comparing the doxorubicin plus trabectedin combination versus doxorubicin alone in first-line therapy in metastatic leiomyosarcoma are pending.

### Combination Therapy With Radiotherapy in Metastatic STS

Spanish groups assessed the combined use of trabectedin and radiotherapy in patients with metastatic STS as phase I/II clinical trial (ClinicalTrials.gov Identifier; NCT02275286) (Martin-Broto et al., 2020). Trabectedin was administered every 3 weeks in a 24-h infusion. Radiotherapy (3 Gy/day for 10 days) was required to start within 1 h after completion of the first trabectedin infusion. In phase 1, recommended dose of trabectedin for this combination treatment was 1.5 mg/m2. In phase 2, among 25 patients, the overall response rate was 72% for local assessment and 60% for central assessment. Overall response rate was calculated as the proportion of patients who achieved a partial or complete RECIST response during therapy.

### Clinical Trial as Neoadjuvant Chemotherapy

One phase II clinical trial in the neoadjuvant setting in patients with advanced localized myxoid liposarcoma has been previously reported ClinicalTrials.gov Identifier; NCT00579501) (Gronchi et al., 2012). The treatment consisted of trabectedin 1.5 mg/m^2^ given as 24-h IV infusion every 3 weeks. Twenty-nine patients received a minimum of three and a maximum of six cycles before surgery. Of 23 patients who could be evaluated by the pathological response, three patients achieved a pathological complete response. Another 12 of 23 had at least a good regression rate (>50% regression). Of 29 patients, seven patients (24%) had a partial response and 21 patients had SD according to the RECIST criteria. One patient died prior to the evaluation due to rhabdomyolysis with hepatic and renal failure after the second trabectedin cycle.

In 2017, phase III clinical trial for evaluating the superiority of neoadjuvant chemotherapy of histotype-tailored regimen to standard chemotherapy (ISG-STS 1001, ClinicalTrials.gov Identifier; NCT01710176) (Gronchi et al., 2017). The STS was non-metastatic, high-risk (high malignancy grade, 5 cm or longer in diameter, and deeply located according to the investing fascia) at extremities or trunk wall and belonging to one of five histological subtypes: high-grade myxoid liposarcoma, leiomyosarcoma, synovial sarcoma, malignant peripheral nerve sheath tumor, and undifferentiated pleomorphic sarcoma. Trabectedin (1.3 mg/m^2^ via 24-h IV infusion) was administered in patients with high-grade myxoid liposarcoma. Patients were randomly assigned (1:1) to receive three cycles of full-dose standard chemotherapy (epirubicin 60 mg/m^2^ per day [short infusion, days 1 and 2] plus ifosfamide 3 g/m^2^ per day [days 1, 2, and 3], repeated every 3 weeks) or histotype-tailored chemotherapy. In the exploratory subgroup analyses according to histology, the difference in disease-free survival favoring standard chemotherapy was consistently seen in all strata, with the exception of high-grade myxoid liposarcoma, in which disease-free survival in the two groups were similar (HR, 1.03; 95%CI, 0.24–4.39).

In addition to previous studies (Gronchi et al., 2016.; Tanaka et al., 2019), the results of clinical trials suggested that preoperative chemotherapy with anthracycline and ifosfamide might be highly effective for treating high-risk STS.

### Safety (Table 2)

Trabectedin was well tolerated in a phase III randomized clinical trial (ET743-SAR-3007, ClinicalTrials.gov Identifier; NCT01343277) (Demetri et al., 2016). The most frequently reported grade 3/4 adverse events were neutropenia (37%) and elevated serum levels of AST/ALT (13%/26%). Less often, grade3/4 creatine phosphokinase elevations (5.3%) and rhabdomyolysis (1.2%) were seen. Deaths associated with drug-related adverse events were infrequent (2.1%). These events were consistent with the well characterized safety and toxicity profiles of trabectedin (Le Cesne, et al., 2013).

**TABLE 2:** Safety profile of trabectedin (NCI-CTC Grade3 or 4 toxicity).

	Demetri et al. (2016)	Le Cesne et al. (2013)
Adverse events	n = 340 (%)	n = 350 (%)
Neutropenia	37	47.6
ALT elevation	26	44.6
Thrombocytopenia	17	13.5
Anemia	14	12.3
AST elevation	13	34.4
Fatigue	6	8.3
CPK elevation	5.3	4.1
Nausea	5	6.3
Vomiting	5	5.1
Rhabdomyolysis	1.2	

Abbreviations: ALT, alanine aminotransferase; AST, aspartate aminotransferase; CPK, creatine phosphokinase; NCI-CTC, National Cancer Institute Common Toxicity Criteria.

The subgroup analysis of the elderly population of ET743-SAR-3007 showed tolerability of trabectedin in elderly patients (Jones et al., 2018). The safety profile for elderly trabectedin-treated patients was comparable to that of the overall trabectedin-treated study. Among 94 patients, the most frequently reported grade 3/4 adverse events were neutropenia (40%) and elevated serum levels of AST/ALT (15%/24%). No unique or unexpected adverse events were noted.

Transaminase increase was the most frequent cause of dose reductions (Calvo et al., 2018). The post hoc analyses of ET743-SAR-3007 confirmed that transaminase elevations were typically highest in the first 2 cycles and mostly transient, non-cumulative, and without clinical consequences, even in patients with grade3/4 transaminase elevations (Calvo et al., 2018). These liver laboratory abnormalities could be managed through dose reduction and delays.

A recurring pattern was observed with increased transaminase levels, typically reaching a peak between days 5 and 7 of each cycle and resolving to grade ≤1 by day 15 without implication for the patient (Brodowicz. 2014). Steroid pretreatment is an effective way of reducing the extent of hepatotoxicity, and steroids are now given routinely before trabectedin administration. Premedication with 20 mg of dexamethasone IV 30 min prior to trabectedin was shown to provide hepatoprotective effects beyond its antiemetic effect (Grosso et al., 2006; Amart et al., 2015).

### Prognostic Factors for the Treatment of Trabectedin

Previous *in vitro* studies have demonstrated that trabectedin cytotoxicity depends on the status of both NER and HR DNA repair pathway (Soares et al., 2007; Italiano et al., 2011; Schoffski et al., 2011; Laroche-Clay et al., 2015). Moreover, DNA-damage biding proteins, which are known components of NER pathway have been described to be a part of the CUL4A ubiquitin ligase complex (Iovine et al., 2011; Moura et al., 2020). The expression of CUL4A could be an indicator of NER pathway integrity and trabectedin efficacy. One prospective translational analysis was performed as a correlative study within the comparative phase II trial that compared trabectedin plus doxorubicin versus doxorubicin alone as first line of advanced STS (Moura et al., 2020). The cases included for gene (n = 66) and protein expression (n = 85). In the group of trabectedin plus doxorubicin (n = 32), overexpression of CUL4A, ERCC1, and ERCC5 significantly correlated with better median PFS, although BRCA1 expression did not correlated with PFS. None of these genes were statistically significant correlated with OS in trabectedin plus doxorubicin group. Furthermore, in the study of phase IIb trial (41), genotype status was available for 60 patients. There was no significant association between BRCA1 haplotype and PFS (Italiano et al., 2018).

## Conclusion

Trabectedin can be administered effectively to patients, but it is important to note that evidence is available for different types of cancer although belonging to the group of advanced STS. Also, trabectedin can be administered safely to the patients although all evidence is limited and future studies should be necessary.
